# A New High Throughput Screening Platform for Cell Encapsulation in Alginate Hydrogel Shows Improved Hepatocyte Functions by Mesenchymal Stromal Cells Co-encapsulation

**DOI:** 10.3389/fmed.2018.00216

**Published:** 2018-08-09

**Authors:** Valeria Iansante, Anil Dhawan, Fatma Masmoudi, Charlotte A. Lee, Raquel Fernandez-Dacosta, Simon Walker, Emer Fitzpatrick, Ragai R. Mitry, Céline Filippi

**Affiliations:** ^1^Dhawan Lab at Mowat Labs, Institute of Liver Studies, King's College London, King's College Hospital, London, United Kingdom; ^2^Paediatric Liver, GI and Nutrition Centre, King's College London, King's College Hospital, London, United Kingdom

**Keywords:** hepatocytes, mesenchymal stromal cells, alginate, hydrogel, microbeads, high throughput screening, acute liver failure, regenerative medicine

## Abstract

Hepatocyte transplantation has emerged as an alternative to liver transplant for liver disease. Hepatocytes encapsulated in alginate microbeads have been proposed for the treatment of acute liver failure, as they are able to provide hepatic functions while the liver regenerates. Furthermore, they do not require immunosuppression, as the alginate protects the hepatocytes from the recipient's immune cells. Mesenchymal stromal cells are very attractive candidates for regenerative medicine, being able to differentiate into cells of the mesenchymal lineages and having extensive proliferative ability. When co-cultured with hepatocytes in two-dimensional cultures, they exert a trophic role, drastically improving hepatocytes survival and functions. In this study we aimed to (i) devise a high throughput system (HTS) to allow testing of a variety of different parameters for cell encapsulation and (ii) using this HTS, investigate whether mesenchymal stromal cells could have beneficial effects on the hepatocytes when co-encapsulated in alginate microbeads. Using our HTS platform, we observed some improvement of hepatocyte behavior with MSCs, subsequently confirmed in the low throughput analysis of cell function in alginate microbeads. Therefore, our study shows that mesenchymal stromal cells may be a good option to improve the function of hepatocytes microbeads. Furthermore, the platform developed may be used for HTS studies on cell encapsulation, in which several conditions (e.g., number of cells, combinations of cells, alginate modifications) could be easily compared at the same time.

## Introduction

Although liver transplantation represents the treatment of choice for patients with end-stage liver disease and liver-based metabolic disorders, in the last three decades human hepatocyte transplantation has emerged as a potential alternative (1–5). Cell transplantation offers several advantages over liver transplantation, being less invasive and readily available, as the cells can be cryopreserved. It also increases the number of recipients who could benefit from the same donor liver, as it is estimated that a cell number equivalent to 10–15% of the recipient liver cell mass is sufficient to replace the liver function. Furthermore, native liver remains in place allowing potential regeneration in patients with acute liver failure (ALF), or future gene therapy treatment for patients with genetic diseases (6, 7).

Intraperitoneal transplantation of human hepatocytes encapsulated in alginate microbeads is an attractive option for the management of patients with ALF, as hepatocytes microbeads can provide hepatic functions while the patient's own liver regenerates, with the advantage of not requiring any immunosuppression, as the alginate protects transplanted hepatocytes from the recipient's immune cells (8, 9). The technique has been proven successful in a rat model of acute liver injury (10). However, the scarce availability of good quality human hepatocytes as a result of the frequent long ischemia time of donor tissues, their short life span after isolation, and the damages incurred after cryopreservation represent major limitations for further development of this technique. Therefore, although theoretically readily available for cell transplantation, hepatocytes are not always suitable to this aim as their quality is often sub-optimal.

Mesenchymal stromal cells (MSC) are multipotent cells found within all mammalian supportive stromal tissues, mainly isolated from bone marrow, umbilical cord and adipose tissues, and able to differentiate *in vitro* into mesenchymal tissue cells, i.e., adipocytes, osteoblasts, and chondrocytes (11–13). We and other groups have shown that MSC drastically improve the survival of hepatocytes and their liver-specific functions in standard cell culture conditions (14–16). Therefore, the main aim of this study was to investigate whether the co-encapsulation of human hepatocytes with MSC in alginate microbeads improved hepatocytes viability and functions.

Because alginate microbead encapsulation is a tedious process with very low throughput, this study also aimed at developing a new platform for fast production of cell alginate microdisks, that would eventually allow comparison of numerous encapsulation conditions—cell types, alginate chemistry, alginate combination, etc. This HTS is based on the cross-linking of alginate directly *in situ*, in cell culture wells. Once this HTS was setup, we used it to compare the function of hepatocytes on their own and with MSCs, and validated the results using actual alginate microbeads.

**Figure 1:**
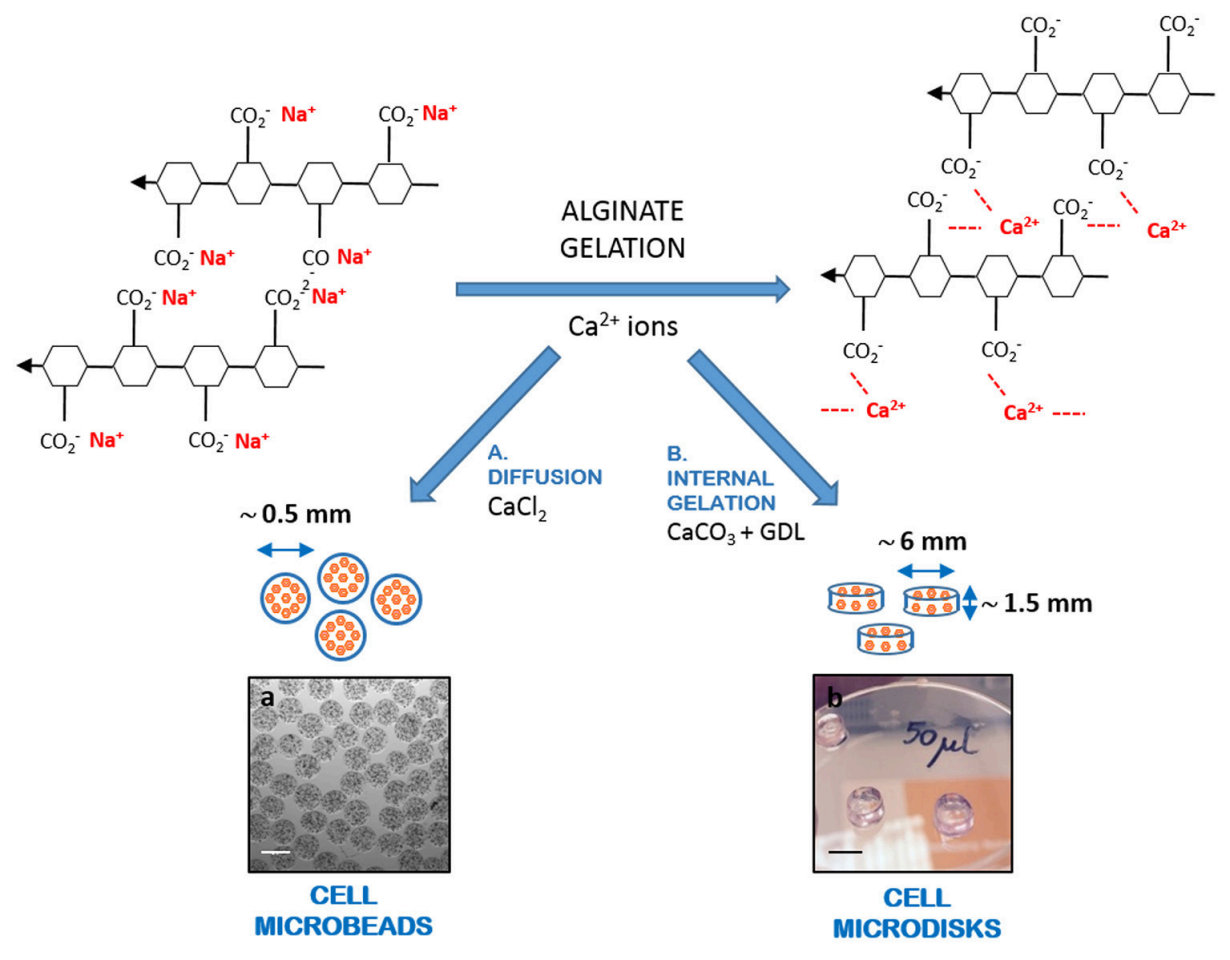
Schematic representation of alginate gelation. Alginate gelation is mediated by the replacement of sodium ions with divalent calcium ions, allowing three-dimensional cross-linking. Gelation can be obtained through **(a)** diffusion, where calcium ions diffuse from a solution containing a highly soluble calcium salt (e.g., CaCl_2_) to the alginate gels; or **(b)** internal gelation, where a low soluble source of calcium (e.g., CaCO_3_) is mixed with the alginate, along with a slow acidifier (e.g., GDL), with production of a gel that assumes the shape of the container where it forms. Gelation by diffusion is used for cell microbeads production, while internal gelation is proposed here for the production of alginate microdisks. Scale bars: **(a)** 500 μm; **(b)** 5 mm.

Alginate, or alginic acid, is a polysaccharide polyanionic linear co-polymer containing blocks of (1,4)-linked β-D-mannuronic (M block) and α-L-guluronic (G block) acids (17–19). Alginate is usually available as sodium-alginate, a sodium salt of alginic acid, where the carboxyl ends of G and M blocks are ionically bound to sodium. Alginate gelation process is done by the addition of divalent cations, such as calcium ions, which replace sodium ions and take part in the interchain ionic binding between G blocks, or cross-linking, with formation of a three-dimensional network (Figure 1). Alginate has been widely used as a biomaterial in cell encapsulation for regenerative medicine applications, as it is highly biocompatible and has low immunogenic properties (20–23). Two main methods can be used for alginate ionic cross-linking, diffusion and internal gelation. The diffusion is mediated by a rapid transfer of gelling ions (e.g., Ca^2+^) from a surrounding solution into the polymer network. It is used when cells are encapsulated in alginate microbeads using an encapsulator, such as the Buchi encapsulator. In this process, cell-alginate droplets are formed in the encapsulator and fall into a CaCl_2_ bath, where they instantaneously crosslink, forming a gel, through the diffusion of calcium ions into the droplet (9, 17). Conversely, the internal gelation is obtained when a low soluble source of divalent ions, such as calcium carbonate (CaCO_3_), is directly mixed with an alginate solution into which the calcium ions are released by addition of a slow acidifier, e.g., D-glucono-δ-lactone (GDL). As GDL is a proton donor and CaCO_3_ a 2-protons acceptor, the adjustment of the molar ratio at 1:2 (CaCO_3_:GDL) allows to keep a neutral pH, compatible with cell encapsulation (24–29).

Although the gelation based on diffusion is extremely fast, the production of alginate microbeads using an encapsulator can be very time-consuming, as the main chamber must be sterilized before every production (9), and replaced for each experimental condition variation. By contrast, the internal gelation approach, despite being slower than the diffusion, allows a direct *in situ* cross-linking of cell-alginate suspension and has the great potential of providing a high throughput screening (HTS) platform, allowing a rapid and parallel testing of different conditions at the same time, thus saving time when a number of encapsulation conditions are compared.

Therefore, the second aim of this study was to investigate whether this new platform for cell encapsulation in alginate, based on internal gelation with production of microdisks, could provide similar results to those obtained by cell encapsulation in alginate microbeads and be used for a variety of cell function analysis.

We first showed that the new proposed HTS platform was able to detect trends seen in the microbeads, as encapsulated hepatocytes in alginate microdisks, showed a similar viability and function variation over time. We then used the new platform to study the effects of co-encapsulation of hepatocytes and mesenchymal stromal cells in alginate microdisks and we found that hepatocytes functions were partially improved by MSC addition. To validate these results, we encapsulated hepatocytes with or without MSC in alginate microbeads. We found that all the hepatic functions analyzed were significantly enhanced by MSC co-encapsulation, confirming the results observed in alginate microdisks and further supporting the use of our HTS platform as a reliable method for the initial pre-screening of encapsulation conditions.

## Materials and methods

### Human cell isolation

All human tissues were approved for research use in accordance with the Research Ethics Committee of King's College Hospital. Written informed consent was obtained from donor relatives or patients.

Human hepatocytes (HC) were isolated from donor liver tissues rejected or unused for orthotopic liver transplantation. Isolation of human hepatocytes was carried out using a modified collagenase perfusion technique (30). Briefly, major hepatic vessels were cannulated and perfused with Hank's buffered salt solution (HBSS, Lonza) containing 0.5 mM ethylene glycol-bis(2-aminoethylether)-N,N,N′,N′-tetraacetic acid (EGTA, Sigma Aldrich) and 4.6 mM 4-(2-Hydroxyethyl)piperazine-1-ethanesulfonic acid (HEPES, Sigma Aldrich). The liver was then flushed with plain HBSS to remove any residue of EGTA. Finally, the tissue was perfused with Eagle's minimum essential medium (EMEM, Lonza) containing 0.05% (w/v) of collagenase P (Roche) at 37°C. Once digested, the tissue was minced and sieved. Hepatocytes were purified by washing 3 times in ice-cold EMEM and centrifuged at 50 g at 4°C for 5 min. Cell number and viability were determined by trypan blue exclusion test. Cells were cryopreserved in University of Wisconsin solution (Bridge to Life) with 5% (w/v) glucose and 10% (v/v) dimethylsulfoxide (DMSO, Sigma Aldrich), using a controlled rate freezer (Kryo 10, Planer Products) and stored at −140°C for later use.

Mesenchymal stromal cells (MSC) were isolated from the Wharton's jelly (WJ), by mechanical dissection, as already described (31), with minor modifications. Briefly, cords from C-sections were obtained from the Anthony Nolan Trust and collected in phosphate buffered saline (PBS, Gibco) with 40 μg/mL of gentamicin (Sigma Aldrich). Cords were cut into sections to expose the WJ, which was minced into very fine pieces (1–3 mm^2^). The explants were plated on sterile petri dishes, allowed to adhere for up to 5 min and covered with MSC culture medium, Minimum Essential Medium α (MEMα Life Technologies), 5% (v/v) platelet lysate (Stemulate Cook Regentec), 40 μg/mL of gentamicin. Cord samples were then incubated at 37°C, 5% CO_2_, and medium half replaced every 3-6 days. Gentamicin was used in the MSC medium for the first 1–2 weeks of culture and then replaced by penicillin/streptomycin (100 U/mL and 100 μg/mL, respectively). Once cell cultures were established, they were expanded, quality controlled, and cryopreserved in MEMα containing 4% (w/v) human albumin (Zenalb®20) and 10% (v/v) DMSO. Cells were positive for stem cells markers (i.e., CD73, CD90, and CD105, expression >75%) and negative for hematopoietic markers (i.e., CD14, CD34, and CD45, <2%) (32).

### Encapsulation of cells in alginate microbeads or microdisks

Human hepatocyte were encapsulated with or without mesenchymal stromal cells in alginate microbeads (MB) using an IE-50R encapsulator (Inotech Encapsulation AG, Dottikon, Switzerland) as previously described (10, 15). Briefly, ultra-pure sodium alginate (PRONOVA SLG20; NovaMatrix, Sandvika, Norway) was dissolved in 0.9% (w/v) NaCl (sterile saline, Baxter) to give a final concentration of 1.5% alginate solution (w/v) and mixed with cells. Microbeads were produced with a 250 μm nozzle, polymerized in 100 mM CaCl_2_ solution for 10 min and washed twice with 0.9% (w/v) NaCl to remove excess Ca^2+^ ions_._

To prepare “*in situ*” cross-linked alginate microdisks (MDs), an internal gelation strategy with some modifications was used (25). Briefly, alginate solution in 0.9% NaCl was mixed with cells (same density as in the microbeads) in the presence of an aqueous suspension of CaCO_3_ (Sigma Aldrich) and D-glucono-delta-lactone (GDL, Sigma Aldrich). The pH was kept neutral by adjusting the molar ratio of CaCO_3_/GDL and the final alginate concentration was 1.5% (w/v). Two different concentrations of calcium carbonate were tested (44 and 52 mM, for microdisks 1 and 2, respectively). Polymerization was obtained at 37°C for 20 min.

The same volume of microbeads/microdisks (50 μL) was used in each well of a 96-well microplate.

### Cell microbeads/microdisks culture

Microbeads and microdisks were cultured in culture medium consisting of Williams E (Sigma Aldrich) supplemented with 10% (v/v) heat-inactivated fetal calf serum (GE Healthcare Hyclone), 2 mM L-glutamine (Gibco), 10 mM Hepes (Gibco), 10 mg/L insulin, 5.5 mg/L transferrin, 670 μg/L sodium selenite (ITS, Gibco), 10^−7^ M dexamethasone (Sigma Aldrich), 100 U/mL penicillin, and 100 μg/mL streptomycin (Gibco). Fifty microliters of MB or MD were maintained in 200 μL of culture medium per well of a 96-well plate in a humidified incubator at 37°C and 5% CO_2_. Medium was replaced every 2–3 days.

Images on microbeads were taken using an inverted microscope (Leica Microsystems Ltd., Milton Keynes, UK).

### Assessment of cell viability and total protein content

Cell viability was determined using a calcein-AM assay. Briefly, supernatant was removed and microbeads or microdisks were depolymerized *in situ* by adding 200 μL of 50 mM ethylenediaminetetraacetic acid (EDTA, Sigma Aldrich), as previously described (23). Dissolution of alginate microbeads or microdisks was allowed by incubation with EDTA for 5 min at 37°C. Cells were recovered by centrifugation, resuspended in 100 μL of PBS and transferred in different wells of a black 96–well plate. Immediately before the detection, 100 μL of 4 μM calcein-AM (Santa Cruz) in PBS was added per well. As calcein-AM penetrates the cells and is converted into green fluorescent calcein by cellular esterases present only in viable cells, cell viability was assessed by measuring the green fluorescence emitted by the cells every minute for 30 min using FLUOstar Omega plate reader (BMG Labtech, Aylesbury, United Kingdom) with an excitation wavelength of 495 nm and an emission wavelength of 515 nm. Results were calculated as relative fluorescence units (RFU)/minute and represented as a percentage over baseline at day 0.

For total protein content measurement, microbeads, or microdisks were depolymerized as described above. Released cells were collected by centrifugation and lysed using a modified lysis buffer, as previously described (50 mM Tris-HCl pH 7.5, 100 mM NaCl, 50 mM NaF, 1 mM Na_3_VO_4_, 30 mM sodium pyrophosphate, 0.5% NP-40, and 0.5 mM PMSF, Sigma Aldrich) (33). Cells lysate were centrifuged at 14,000 rpm for 10 min to remove cell debris. Quantitation of total protein was performed by using bicinchoninic acid (BCA) Protein Assay Kit (Pierce^TM^), according to the manufacturer's instructions.

### Tests of hepatocyte-specific functions

Human albumin and alpha1-antitrypsin secreted in the culture medium over 24 h by microbeads or microdisks and/or released in rat plasma were quantified using a human albumin/alpha1-antritrypsin Enzyme Linked Immunosorbent Assays (ELISA). Briefly, an ELISA plate was coated with 10 μg/ml of goat anti-human albumin antibody or 10 μg/ml of goat anti-human alpha1-antitrypsin (Bethyl Laboratories, Tx, USA) in coating buffer (50 mM carbonate-bicarbonate, pH 9.6, Sigma Aldrich) for 1 h, washed with wash buffer (50 mM Tris, 140 mM NaCl, 0.05% Tween 20, pH 8.0, Sigma Aldrich) and blocked with blocking buffer (1% bovine serum albumin, 50 mM Tris, 140 mM NaCl, pH 8.0, Sigma Aldrich) for 30 min. One hundred microliters of standards (400–6.25 ng/L) or samples were added and incubated at room temperature for 1 h. The plate was washed and a Horseradish Peroxidase (HRP)-conjugated goat anti-human albumin antibody or anti-human alpha1-antritrypsin antibody (1 mg/mL, Bethyl Laboratories, Tx, USA) added at a 1:150,000 or 1:50,000 dilution, respectively, in diluent (50 mM Tris, 140 mM NaCl, 1% bovine serum albumin, 0.05% Tween 20, pH 8.0, Sigma Aldrich) for 1 h. 3,3′,5,5′-tetramethylbenzidine (TMB) substrate solution (Insight Biotechnology) was added and incubated in the dark for 10 min. The reaction was stopped with 2M H_2_SO_4_. The optical density was measured at λ = 450 nm on a FLUOstar Omega plate reader.

For urea synthesis measurement, microbeads or microdisks were washed twice with PBS and incubated with 5 mM ammonium chloride in serum-free medium for 6 h at 37°C, 5% CO_2_. The supernatant was collected and analyzed using a QuantiChrom Urea Assay Kit (Universal Biologicals, Cambridge, UK), according to the manufacturer's instructions.

### *In vivo* studies

Sprague Dawley male rats 8–10 weeks old and weighing between 200 and 300 g were used in this study. Animals were maintained in conventional housing facilities and received standard care. The experiments were performed after acclimatization for at least 7 days.

Rats were transplanted intraperitoneally with alginate microbeads containing either HC alone or co-encapsulated with MSC, or transplant medium (sham) (*n* = 3 each group). The microbeads were produced as described above and resuspended in transplant medium (CMRL) at a ratio 2:1 (microbeads:CMRL). Each animal received microbeads suspension at a dose of 10 ml/kg.

Blood sampling was performed at days 1, 3, and 7 after transplantation and plasma obtained through centrifugation at 2,000 × g for 15 min. As human cells were used, human albumin and human alpha1-antitrypsin released in the plasma were analyzed (ELISA assay, see above) as an indirect method to detect microbeads performance.

### Statistical analysis

Statistical analyses were performed using GraphPad Prism 7.01 software (GraphPad, CA, USA). Differences between groups were assessed using two-way ANOVA with Sidak (2 groups) or Tukey (>2 groups) multiple testing correction. Differences were considered statistically significant when *p*-values were lower than 0.05. All data are presented as mean values ± standard error of the mean deviation (SEM).

## Results

### Comparison of alginate microbeads and microdisks for hepatocyte encapsulation

A preliminary study was performed to identify the best conditions to crosslink the alginate *in situ*, directly in the wells of a 96-well plate. To this aim, alginate was mixed with increasing concentrations of CaCO_3_ (from 20 to 60 mM) and the gelling process was triggered by addition of GDL. The microdisks were observed for up to 10 days and assessed for their ability to efficiently keep an even shape, without showing any obvious signs of shrinkage. The best CaCO_3_ concentrations allowing the production of stable microdisks were 44 and 52 mM, hereafter named MD_1_ and MD_2_, subsequently tested for cell encapsulation. Lower concentration of CaCO_3_ resulted in the formation of microdisks that dissolved over time.

**Figure 2:**
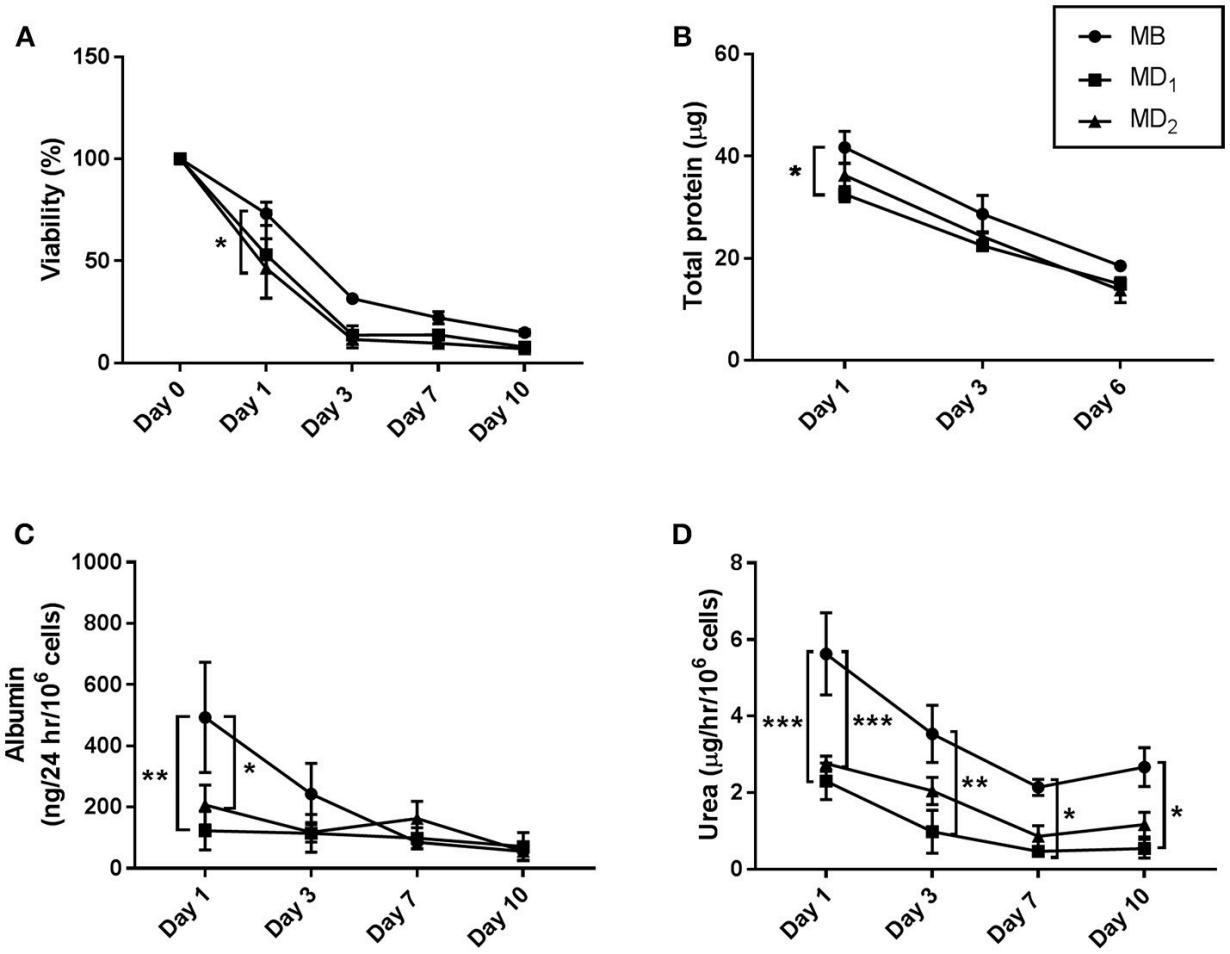
Viability, total protein content, albumin and urea production of hepatocytes encapsulated in alginate microbeads (MB) or microdisks (MD), containing 44 mM (MD1), or 52 mM (MD2) of CaCO3. **(A)** Cell viability measured by calcein-AM assay and expressed as % over baseline value at day 0. Cells were released from the alginate by incubation of microbeads/microdisks with 50 mM EDTA. **(B)** Total protein content was assessed after alginate microbeads/microdisks depolymerization as above. **(C)** Albumin synthesis was measured by ELISA from microdisk/microbead supernatants. **(D)** Urea production measured on disk/bead supernatants following incubation with 5 mM ammonium chloride for 6 h. *N* = 3. Data represent mean ± SD. *p < 0.05; **p ≤ 0.01; ***p ≤ 0.001.

Primary human hepatocytes were then encapsulated in alginate microdisks (MD_1_ or MD_2_) or microbeads (MB). Cell viability was similar in all the conditions tested at different time points, except at day 1 when hepatocytes in MD_2_ showed a significant lower viability (46.4 ± 14.5%) than in MB (73.2 ± 5.7, *p* ≤ 0.05) (Figure 2A). Likewise, the total protein content (TP) measured on hepatocytes encapsulated in microbeads or microdisks was similar, showing a significant difference only at day 1, when cells were encapsulated in MD_1_ (TP = 32.6 ± 1.5 μg, *p* < 0.05), but not in MD_2_ (TP = 36.3 ± 2.4 μg), compared to MB (TP = 41.7 ± 3.2 μg) (Figure 2B).

The ability of hepatocytes to produce albumin and urea was tested as a direct method to measure hepatocytes-specific functions. Albumin assay showed significant differences between MB and MD at day 1, when hepatocytes encapsulated in MB released higher amount of albumin (493 ± 180 ng/24 h/10^6^ cells) than in MD_1_ (123 ± 63 ng/24 h/10^6^ cells, *p* ≤ 0.01) and MD_2_ (207 ± 65 ng/24 h/10^6^ cells, *p* ≤ 0.05). However, a similar amount of albumin was released in the different conditions at later time points (Figure 2C). Similarly, the production of urea was significantly decreased at day 1 in MD_1_ and MD_2_ (2.3 ± 0.5 and 2.8 ± 0.2 μg/h/10^6^ cells, respectively) compared to MB (5.6 ± 1.1 μg/h/10^6^ cells, *p* ≤ 0.001). Whilst the urea produced by hepatocytes encapsulated in MD_1_ was significantly lower than in MB in all the time points analyzed, no significant difference was observed in MD_2_ at late time points (Figure 2D).

### Co-encapsulation of human hepatocytes and mesenchymal stromal cells in alginate microdisks

As the function of hepatocytes appeared most similar to microbeads in the MD_2_ conditions, we decided to use these microdisks as a new platform for direct *in situ* alginate cross-linking to investigate the effect of mesenchymal stromal cells (MSC) co-encapsulation with hepatocytes (HC).

**Figure 3:**
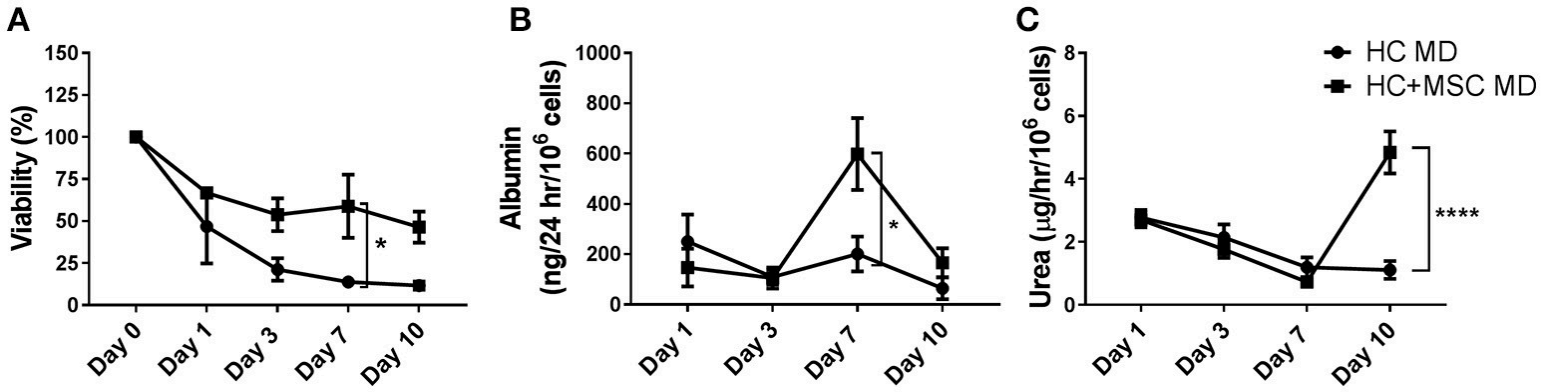
Viability, albumin and urea production of hepatocytes (HC) encapsulated in alginate microdisks with and without mesenchymal stromal cells (MSC). **(A)** Cell viability measured by calcein-AM assay and expressed as % over baseline value at day 0. Cells were released by incubation of microdisks with EDTA. **(B)** Albumin synthesis measured by ELISA on cell supernatants. **(C)** Urea production measured using a colorimetric assay on cell supernatant following incubation with 5 mM ammonium chloride for 6 h. Ureogenesis is expressed as μg of urea released in 1 h by 10^6^ cells. *N* = 3. Data represent mean ± SEM. *p < 0.05; ****p ≤ 0.0001.

Cell viability was expressed as percentage of the baseline value at day 0, in order to compare microdisks containing HC with or without MSC. Both groups showed similar viability, except at day 7, when HC co-encapsulated with MSC showed higher viability than HC alone (13.9 ± 1.7 vs. 58.9 ± 18.8% for HC and HC+MSC microdisks, respectively, *p* ≤ 0.05) (Figure 3A).

Similarly, albumin production was significantly improved when HC were co-encapsulated with MSC 7 days after encapsulation (201 ± 69 vs. 598 ± 143 ng/24 h/10^6^ cells for HC and HC+MSC microdisks, respectively, *p* ≤ 0.05) (Figure 3B). Interestingly, urea production was significantly improved in the co-encapsulated cells only later, at day 10 (1.1 ± 0.3 vs. 4.8 ± 0.7 μg/h/10^6^ cells, *p* ≤ 0.0001) (Figure 3C).

### Co-encapsulation of human hepatocytes and mesenchymal stromal cells in alginate microbeads

**Figure 4:**
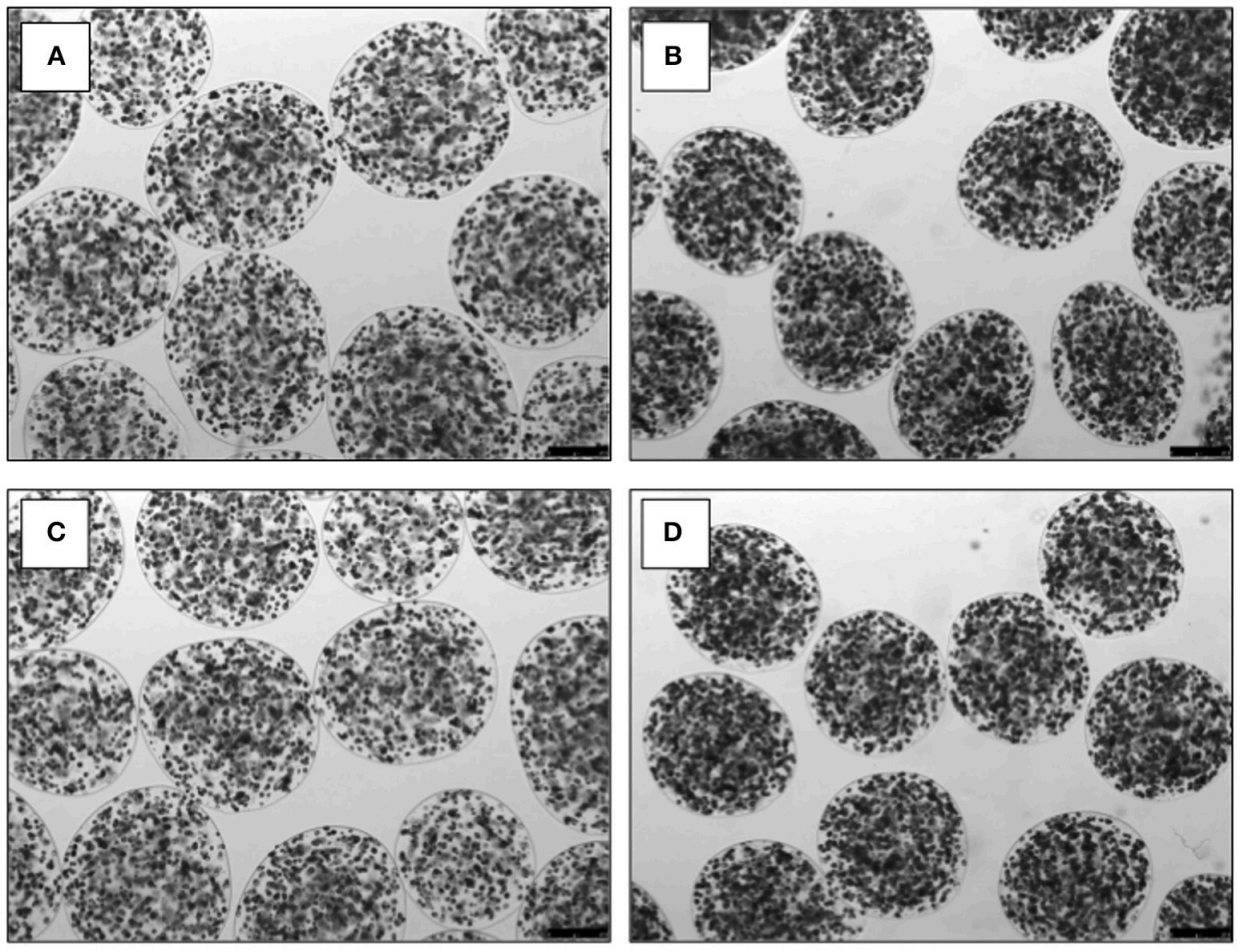
Images of alginate microbeads containing hepatocytes **(A,C)** or hepatocytes and mesenchymal stromal cells **(B,D)**. Images on microbeads were taken at day 1 **(A,B)** and day 7 **(C,D)**, using an inverted microscope (Leica Microsystems Ltd., Milton Keynes, UK). Scale bar: 250 μm.

As albumin and urea production were significantly improved when HC were co-encapsulated with MSC in alginate microdisks, we decided to produce alginate microbeads containing either HC or HC+MSC, to see whether these results could be validated. The alginate microbeads obtained using HC with or without MSC were similar in shape and size and did not show any signs of shrinkage over time (Figure 4).

**Figure 5:**
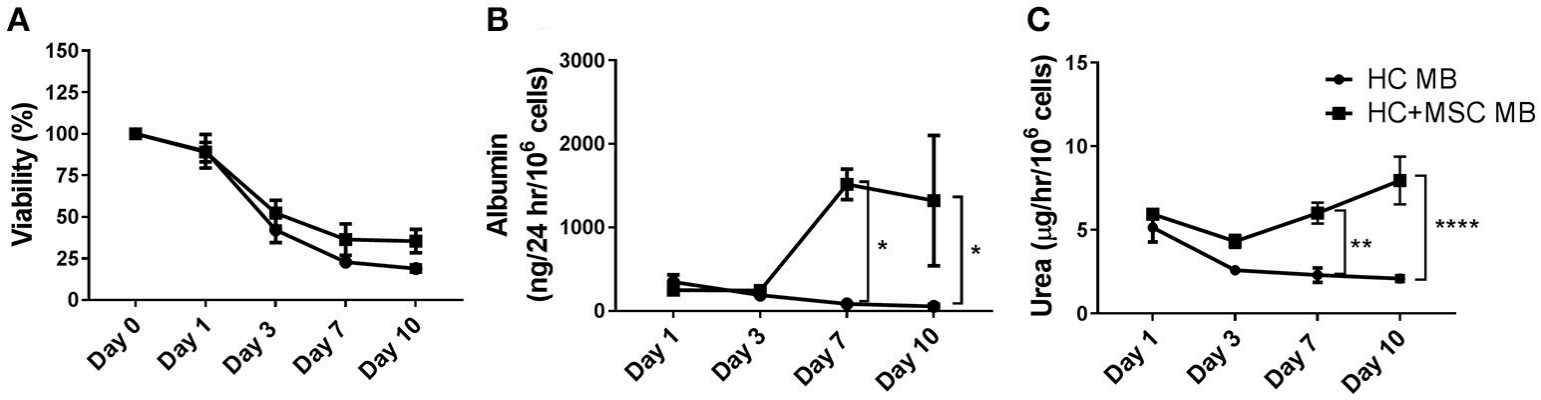
Viability, albumin and urea production of hepatocytes (HC) encapsulated in alginate microbeads with and without mesenchymal stromal cells (MSC). **(A)** Cell viability measured by calcein-AM assay and expressed as % over baseline value at day 0, after release from the alginate. **(B)** Albumin synthesis measured by ELISA on cell supernatants. **(C)** Urea production measured using a colorimetric assay on cell supernatant following incubation with 5 mM ammonium chloride for 6 h. Ureogenesis is expressed as μg of urea released in 1 h by 10^6^ cells. *N* = 3. Data represent mean ± SEM. *p < 0.05; **p < 0.01; ****p ≤ 0.0001.

Cell viability did not show any significant difference in the groups analyzed (Figure 5A). Albumin and urea production were measured to assess hepatocyte-specific functions. Albumin released in the supernatant was significantly increased in the co-encapsulated cells compared to HC alone at day 7 (85 ± 5 vs. 1516 ± 182 ng/24 h/10^6^ for HC and HC+MSC microbeads, respectively, *p* ≤ 0.05) and day 10 (56 ± 28 vs. 1321 ± 780 ng/24 h/10^6^ cells, *p* ≤ 0.05) (Figure 5B). Similarly, urea production was significantly improved by the addition of MSC at late time points (2.3 ± 0.4 vs. 6.0 ± 0.6μg/h/10^6^ cells at day 7, *p* ≤ 0.01, and 2.1 ± 0.2 vs. 8.0 ± 1.4 μg/h/10^6^ cells at day 10, *p* ≤ 0.0001, for HC and HC+MSC microbeads, respectively) (Figure 5C).

### Function of co-encapsulated human hepatocytes and mesenchymal stromal cells microbeads *in vivo*

**Figure 6:**
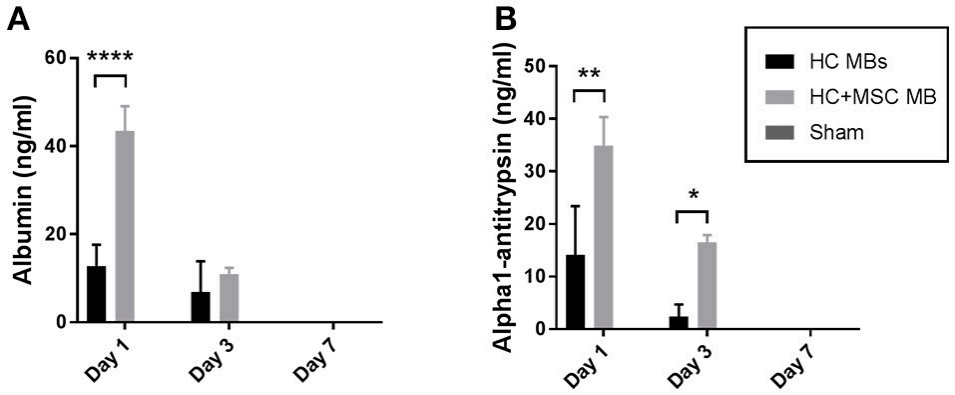
Human liver-specific proteins released in rat plasma after microbeads transplantation. Alginate microbeads containing HC with and without MSC were transplanted intraperitoneally in normal Sprague-Dawley rats. Blood samples were collected at day 1, 3, and 7 and plasma obtained through centrifugation. Human albumin **(A)** and alpha1-antitrypsin **(B)** levels were measured by ELISA. Data represent mean ± SEM. *p < 0.05; **p < 0.01; ****p ≤ 0.0001.

Normal Sprague Dawley rats were transplanted intraperitoneally with alginate microbeads containing either hepatocytes alone or co-encapsulated with MSC. Human cells were used so that the human proteins detected in rat plasma could be used as an indirect measurement of microbeads functions. Plasma samples obtained from the animals were tested for human albumin (Alb) and alpha1-antitrypsin (AAT) levels (Figure 6). Interestingly, both human proteins were significantly higher in the animals transplanted with a combination of HC and MSC compared to HC alone the day after transplantation (Alb: 43.4 ± 5.6 vs. 12.8 ± 4.8 ng/ml, *p* ≤ 0.0001; AAT: 34.9 ± 5.4 vs. 14.1 ± 9.3 ng/ml, *p* ≤ 0.01). As expected, these proteins were absent in the control animal transplanted only with transplant medium (sham). Three days after transplantation there was a decrease in the levels of human proteins in rat plasma, with significant differences between the groups analyzed only for alpha1-antitrypsin (2.4 ± 2.4 vs. 16.5 ± 1.4 ng/ml, *p* ≤ 0.05, in HC vs. HC+MSC microbeads transplanted groups, respectively). The human proteins became undetectable at day 7.

## Discussion

Acute Liver Failure (ALF) has a high mortality risk in patients listed for liver transplantation not receiving an allograft (34). Transplantation of human hepatocytes encapsulated in alginate microbeads (MB) represents a promising alternative to liver transplantation, as cells provide missing hepatic functions while the patient's own liver regenerates. Furthermore, the presence of alginate allows protection of the hepatocytes from immune cells, thus not requiring immunosuppression after transplantation. Human primary hepatocytes are isolated from donor livers that are unused for liver transplantation and offered for cell isolation. Unfortunately, these marginal livers frequently have long ischemia time, high content of fats, giving rise to hepatocytes of poor quality and with limited survival (35). An ideal treatment of ALF with encapsulated human hepatocytes should provide hepatic functions for several days, thus allowing the regeneration of the native liver. We tried to address this issue co-encapsulating human hepatocytes (HC) with mesenchymal stromal cells (MSC), knowing that MSC are able to improve HC functions when co-cultured in 2-D cell cultures, as already shown by us and other groups (15).

Although the encapsulation of cells in alginate microbeads is a relatively quick process, it requires prior sterilization of the main chamber containing the microbeads, thus making the whole technique very time-consuming when a number of different conditions are compared. An ideal platform to test a multitude of encapsulation conditions at the same time may be represented by thin layer microdisks made of alginate produced directly in cell culture plates. Unfortunately, it is not possible to prepare alginate microdisks by simply adding CaCl_2_ to an alginate solution in a cell culture well, as the very rapid binding of calcium ions to the G blocks of the alginate determines the formation of uneven disks. Galateanu et al. were able to prepare thin alginate layers directly in culture dishes, by placing the alginate solution between two filter papers soaked in CaCl_2_ or calcium gluconate solution. The release of calcium ions from the paper was sufficiently slow to allow the production of even microdisks (36). Although effective, this method was not compatible with the development of a high throughput screening platform, as it was time-consuming and required the removal of filter papers from each disk after production to allow exchanges of gases, nutrients and cell products, between the gels and cell culture medium.

We decided to test a different approach for HTS cell encapsulation: *in situ* cross-linking the alginate in cell culture wells via internal gelation, using CaCO_3_ and GDL. Internal gelation has been already proven safe for cell encapsulation, having been proposed for regenerative medicine applications, for instance for cell delivery in tissue regeneration therapy when a delayed *in situ* crosslinking is needed, e.g., bone regeneration applications (37, 38). Nevertheless, this approach was never tested for HTS applications with the aim of screening cell encapsulation conditions to use for cell microbeads production. In other words, the two main methods for ionic alginate gelation, i.e., internal gelation and ions diffusion, have never been compared to see whether they could support encapsulated cells to a similar extent.

Such a comparison is essential, considering that the internal structure of alginate microbeads and microdisks is very different. The microbeads, produced by ions diffusion, exhibit an inhomogeneous alginate distribution, as the alginate concentration gradually decreases toward the center of the gel. By contrast, the microdisks, obtained by internal gelation, have a homogenous structure as the concentration of calcium is even throughout the gel. Furthermore, the size of the microbeads and microdisks is very different, the volume of each microdisk being more than 50 times larger than the microbead. Overall, we observed that hepatocytes encapsulated in microbeads performed better than in microdisks in terms of viability, total protein content and main hepatic functions. Between the two microdisks polymerization conditions used, MD_2_ showed better cell functions than what was observed in MD_1_. We believe that the optimization of microdisks encapsulation conditions is paramount when this approach is proposed for HTS applications. In fact, different alginates with specific molecular weight and G/M composition may require fine adjustments of CaCO_3_ concentration.

Since hepatocytes encapsulated in alginate microdisks MD_2_, although inferior to microbeads the day after encapsulation, showed a similar trend in terms of functions at later time points, we decided to use the microdisks to study the effect of the co-encapsulation of hepatocytes with mesenchymal stromal cells. Our results indicated that cell viability, albumin production and ureogenesis were partially improved by the addition of MSC (Figure 3). However, when the co-encapsulation was tested in alginate microbeads, hepatocyte ability of producing albumin was not just improved by MSC, as seen in the microdisks, but also increased over time, in a way that 1 week after production beads containing HC+MSC were able to synthesize more than 10 times the amount of albumin produced at day 1. A similar trend was observed in a previous study (15), where 2-D co-cultures of HC with MSC resulted in more than 10-fold increased albumin starting at day 7 and keep increasing up to day 15. Whether this effect is due to the potential of MSCs to differentiate into hepatocytes or to their stimulatory/trophic effects is still unclear. Furthermore, hepatocytes co-encapsulated with MSC in alginate microbeads produced a higher amount of urea compared to HC on their own. This feature was observed in the microdisks only at day 10, while it was anticipated in the microbeads, starting at day 7. A possible reason for this difference in timing and in the overall lower levels of cell functions observed in the microdisks may be related to a differential cell oxygenation between microbeads and microdisks. Cells encapsulated in the microbeads will be submitted to a lower oxygenation gradient than when embedded in microdisks, due to the smaller size. The lower oxygen tension could affect the cells in a different way when they are encapsulated in microbeads or microdisks, thus explaining the difference observed (39–41).

Although the performance of the cells encapsulated in alginate microdisks did not seem as good as what observed in alginate microbeads, the beneficial effect of MSC addition was detectable in the microdisks too, even though to a less extent to what observed in the microbeads. Hence, for finer comparison, after the initial screening, the production of microbeads was necessary to detect the full extent of differences between the two conditions. This suggests that cell encapsulation in microdisks could be a valuable tool for high throughput pre-screening studies to identify gross differences between the conditions analyzed, such as combinations of cells, as described here in our small scale study, and potentially in larger investigations to compare different alginate modifications (e.g., peptide, microfiber, minerals), to titrate cell density for alginate encapsulation, or testing the encapsulated cells with different compounds. The same testing can be done using microbeads, however the production of microdisks is much faster, thus more compatible with HTS tests.

The improved functions observed *in vitro* were further confirmed *in vivo*. In fact, a higher amount of human liver-specific proteins were detected in the plasma of rats transplanted with HC and MSC co-encapsulated microbeads compared to HC microbeads. However, human albumin and alpha1-antitrypsin were detected only up to 3 days after transplantation. A possible reason for this rapid decline may be related to the fact that human cells were transplanted in a rat recipient. Although immune cells are not able to penetrate the microbeads, the human proteins released can be targeted by the recipient's immunity and destroyed.

Our *in vivo* study was not performed in a model of acute liver failure, as this application was beyond the main scope of our investigation. However, these results warrant the investigation of the therapeutic potential of our co-encapsulated cells microbeads and compare them to other cell microbeads already described in the literature. The co-encapsulation of hepatocytes and mesenchymal stromal cells in alginate microbeads for the treatment of acute liver failure was already proposed by Shi et al. (42), using cells isolated from rats and an encapsulation protocol based on the coating of microbeads with poly-L-Lysine (PLL) and an additional outer layer of alginate, followed by depolymerization of the inner core, with the aim of improving cell-to-cell contact. Although the results were promising, we believe that our approach may offer more realistic clinical applications, as the presence of PLL, potentially immunogenic *in vivo*, is not ideal for the use of microbeads in patients. Also, the depolymerized core possibly allows proliferation of the cells, that, we think, may be dangerous as it could affect the stability of the microbeads. Furthermore, it has been described that PLL is a powerful inhibitor of miRNA biogenesis (43) with pronounced effects on hepatocyte function, such as alteration of the expression of miRNAs involved in the regulation of insulin sensitivity and cell proliferation (44). Meier et al. developed a protocol for the co-encapsulation of hepatocytes and MSC in hybrid poly(ethylene glycol)-alginate hydrogel microbeads, which, unlike poly-L-lysine-based hydrogels, have an excellent biological acceptance (45). However, cells are able to proliferate in these microbeads, facing the same risks described above for the microbeads with depolymerized center.

In conclusion, we have shown in this study that alginate microdisks, produced by internal gelation process, represent a possible first high throughput screening method to analyse and compare several conditions at the same time. Although the functions of cells encapsulated in alginate microdisks were overall lower than what observed in the microbeads, when this method was used to test the co-encapsulation of hepatocytes with mesenchymal stromal cells, it was able to detect improved functions compared to the microdisks containing only hepatocytes. These results were confirmed and further refined when using alginate microbeads. On the basis of the improved functions observed when MSC are co-encapsulated with hepatocytes, it seems that they could represent a valuable tool to improve the efficacy of hepatocyte alginate microbeads for the treatment of acute liver failure, although further experiments are required to fully support this statement.

## Data availability

The raw data supporting the conclusions of this manuscript will be made available by the authors, without undue reservation, to any qualified researcher.

## Author contributions

VI performed the experiments and wrote the first draft of the article. VI, CF, AD, and EF contributed to the conception and design of the study. FM contributed to alginate microdisks characterization. RM, CF, VI, CL, RF-D, and SW isolated primary hepatocytes. VI, RF-D, and SW isolated MSC. All the authors contributed to manuscript revision, read and approved the submitted version.

### Conflict of interest statement

The authors declare that the research was conducted in the absence of any commercial or financial relationships that could be construed as a potential conflict of interest. The reviewer VML and handling Editor declared their shared affiliation.
